# The influence of temperature variation on the levels of the International Dysphagia Diet Standardization Initiative

**DOI:** 10.1590/2317-1782/20242023315en

**Published:** 2024-09-13

**Authors:** Tainá Cristina do Espírito Santo, Adriana Ponsoni, Cinthia Madeira de Souza, Lucia Figueiredo Mourao

**Affiliations:** 1 Faculdade de Ciências Médicas, Universidade Estadual de Campinas – UNICAMP - Campinas (SP), Brasil.; 2 Programa de Pós-graduação em gerontologia, Faculdade de Ciências Médicas, Universidade Estadual de Campinas – UNICAMP - Campinas (SP), Brasil.; 3 Departamento de Desenvolvimento Humano e Reabilitação, Faculdade de Ciências Médicas, Universidade Estadual de Campinas – UNICAMP -Campinas (SP), Brasil.

**Keywords:** Swallowing, Swallowing Disorder, Viscosity, Temperature, Hospitals

## Abstract

**Purpose:**

To analyze the influence of temperature on the flow/texture of different foods, immediately after preparation and after one hour, and 2) To compare the influence of varying the cook in food preparation, in relation to food flow.

**Methods:**

This is a quantitative and experimental study. The IDDSI standardized flow test was used to evaluate the remaining volume in the syringe and the levels of foods (porridge, smoothie, liquid soup, and pureed light soup) prepared by different cooks, in triplicate, at time zero (T0) and after one hour (T1).

**Results:**

Differences in temperature were observed in all foods at T0 and T1 (p < 0.05). The IDDSI level changed only in porridge, from level 3 to 4 (p = 0.043). Modifications were observed in the preparation by different cooks for smoothie, on the 2nd and 3rd day (p = 0.049), from level 3 to 4 of IDDSI. In porridge, on the 1st and 3rd day (p = 0.048) and 2nd and 3rd day (p = 0.048), with a change from level 4 to 3 of IDDSI.

**Conclusion:**

The temperature of all foods differed within the one-hour interval, with modifications in the flow test and in the IDDSI levels, from level 3 to 4, only for porridge. Different cooks prepared the smoothie and porridge with different characteristics, resulting in changes from level 3 to 4 in both foods.

## INTRODUCTION

Swallowing is a complex and vital process that involves correlated structures and neural mechanisms^(1)^. Alterations in the neurophysiology of swallowing lead to dysphagia, characterized by difficulty in preparing a bolus of food or transporting it to the stomach^(2)^. In hospital settings, dysphagia contributes to increased healthcare costs and prolonged hospital stays^(3)^, overburdening the healthcare system^(3)^. The prevalence of dysphagia is notably higher among hospitalized male patients with heart disease and older adults^(4)^, with approximately 50% of the older adult population affected^(5)^. The underlying causes are neurological diseases and age-related changes in the structures involved in swallowing^(6)^, resulting in malnutrition, dehydration, prolonged hospitalization, respiratory complications requiring intubation, drug treatment for aspiration pneumonia, and even death^(5)^.

In hospitals, dysphagia treatment is prioritized to ensure safe and efficient swallowing^(7,8)^. One of the most common therapeutic options is altering the consistency of foods^(8)^ and liquids to facilitate swallowing and reduce the risk of aspiration^(9,10)^. The clinical intervention of food texture modifications^(8)^ has necessitated the establishment of an international standard for classifying food consistency^(11)^. In 2013, the International Dysphagia Diet Standardization Initiative (IDDSI)^(12)^ developed a terminology to classify the texture of food and the thickness of beverages using a diagram (IDDSI diagram) that provides professionals with appropriate indications of food or beverages for individuals with dysphagia. This also promotes efficient communication between speech and language therapists and the multidisciplinary team^(8)^. IDDSI tests consist of simple, and easily accessible tests that should be carried out under conditions in which food and drinks are served (especially temperature)^(9)^. A study described that viscosity and temperature affect food consistency^(13)^. The addition of cornstarch-based thickeners to coffee at 70 ºC results in a thicker sample than that at room temperature (25 ºC), as the cornstarch granules increase the liquid’s viscosity during gelation^(13)^. Therefore, these variables can alter fluid consistency, which may affect swallowing safety in patients with dysphagia^(14)^.

Thus, the hypothesis of this study was that the temperature of food may change from the time of final preparation until it is served to the patient. Our second hypothesis was that different cooks prepare meals differently, affecting the flow of thickened liquids. Therefore, the objectives were: 1) to analyze the influence of temperature on the flow/texture of different foods immediately after preparation and after one hour and 2) to compare the influence of different cooking methods on the flow of food.

## METHOD

### Study structure

This study was conducted using a quantitative methodology and an experimental approach. It is worth mentioning that experimental research is characterized by the direct manipulation of variables associated with the object of study and aims to test hypotheses^(15)^. By manipulating the quantity and quality of the variables, a relationship between cause and effect is established, requiring control of the variables and evaluation of the results of these relationships^(16)^. Therefore, our dependent variables were temperature and the involvement of different cooks in meal preparation.

### Place and period of data collection

Data were collected in August 2022 at the Clinical Hospital of the State University of Campinas (Unicamp). As this was an experimental study without human intervention, it was not necessary to consult an ethics committee.

### Study material

The materials used for data collection included a digital thermometer to measure the temperature of the food, a cell phone camera mounted on a tripod to record all procedures by filming, BD® brand 10 mL syringes (standard for flow tests) and plastic utensils such as spoons and forks.

### Research instruments

#### Flow test

**Figure 1:**
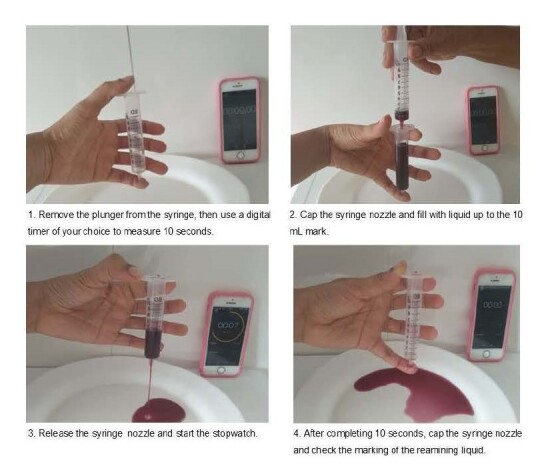
IDDSI flow test

A flow test instrument (Figure 1) was used for data collection. Thickened liquids were filled into a 10 mL BD syringe to the 10 mL mark. The tip of the syringe was then covered with a finger to prevent liquid flow. When the finger was released, 10 s were counted; after the count was completed, the tip was capped (Figure 1). Thus, the amount of liquid remaining in the syringe was determined ^(12)^.

The remaining liquids were classified according to levels 0 to 4 of the IDDSI: level 0, low viscosity; less than 1 ml of liquid remaining in the syringe at the end of the 10 second flow; level 1, slightly viscous, between 1 and 4 ml remaining in the syringe; level 2, slightly viscous, between 4 and 8 ml remaining in the syringe; and level 3, moderately viscous, when more than 8 ml remains in the syringe^(12)^.

Level 4: Extremely viscous. If the flow test was not applicable (10 ml remaining), the spoon tilt test or fork drop and pressure tests were performed^(12)^.

In this study, spoon tipping, fork dripping, and fork pressure tests were performed according to the method validated by the IDDSI^(12)^. In the first test, the spoon was tilted with food dropped onto the surface without leaving any residue on the utensil^(12)^. In the second test, food was placed on a fork and dripping was observed^(12)^. In the last test, pressure was applied to the food using a fork. For classification, it was necessary to observe discrete traces of cutlery^(12)^.

#### Data collection

**Figure 2:**
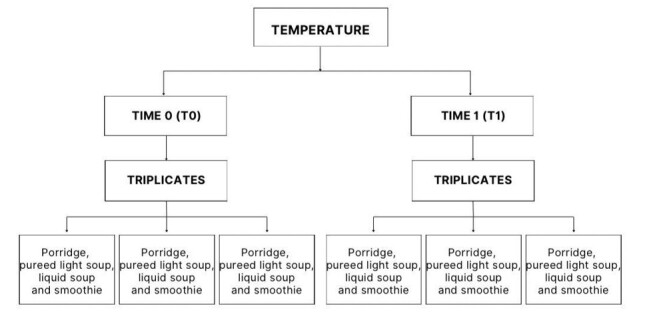
Demonstration of the collection of all foods, in triplicate, at Time 0 (T0) and Time 1 (T1)

**Figure 3:**
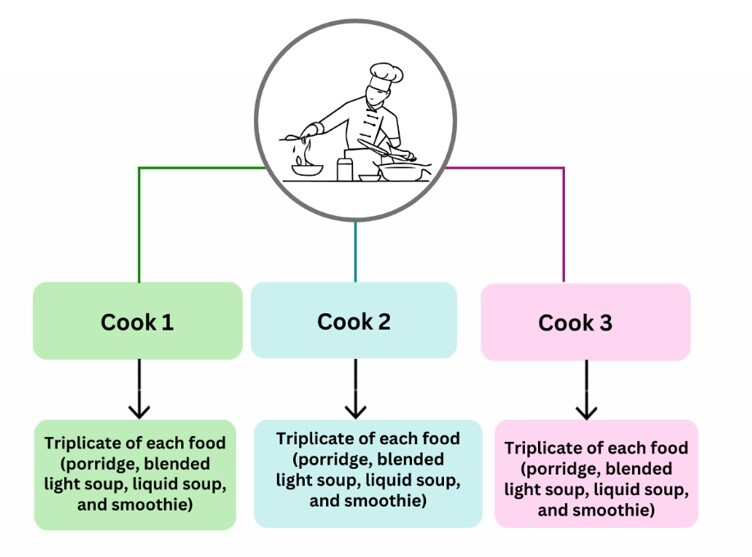
Demonstration of the food collection in triplicate, with variation of the cooks

Data collection for the study was carried out in two stages: the first stage involved testing temperature, and the second stage involved the variation of cooks in food preparation. As this was an experimental study designed to ensure the reliability, precision, and reproducibility of the results, with the aim of minimizing experimental error, data collection in stages 1 and 2 (Figures 2, 3) was performed in triplicate. Each variable that could affect the change in food texture was tested three times.

As the study had an experimental design, one sample of each food tested with the least variation in the dependent variables (temperature and cooking process) was selected.

#### Level 1 - Temperature

During the first stage of the IDDSI flow test, the food temperature was recorded. First, the temperature was measured using a digital thermometer after the preparation of each meal (porridge, pureed light soup, liquid soup, and smoothie) (Chart 1), referred to as time zero (T0), and one hour later, referred to as time one (T1). The one-hour interval was chosen to reproduce the estimated time required for the meal to reach the hospital patient's bedside.

**Chart 1:** Food characterization

**Food**	**Characteristics**	**Ingredients**	**Calories (kcal)**	**Proteins (g)**	**Carbohydrates (g)**	**Lipids (g)**
Porridge	Blended ingredients	milk, sugar and[[Q6: Q6]] maize starch	248.72	8.31	35.09	8.3
Pureed light soup	Blended ingredients	water, carrot, animal protein and seasonings	302.51	16.4	33.78	11.3
Liquid Soup	Pulverized Ingredientsand thicker than water	water, carrot, animal protein and seasonings	302.51	16.4	33.78	11.3
Smoothie	Blended ingredients	milk, banana and redcurrant[[Q6: Q6]]	333.14	8.2	72.2	4.63

**Caption:** Kilocalories (kcal); grams (g)

The temperatures of the foods (porridge, pureed light soup, liquid soup and smoothie) were measured at T0 and T1 (Figure 2), for three samples of each food, i.e. triplicate samples were considered to determine the smallest temperature variation for analysis.

#### Level 2 – Analysis of the different cooks during food preparation

Over a period of three alternate days, foods (porridge, pureed light soup, liquid soup, and smoothies) were collected in triplicate. Alternate days were chosen to include different cooks in the study, as the staff rotated in preparing meals. Therefore, in the second phase of the study, the cooks who prepared meals were considered the dependent variables. (Figure 3)

Notably, various chefs used standardized recipes with identical quantities of ingredients to prepare meals, as determined by the hospital's nutrition team. All the industrially produced ingredients were purchased from the same brand. Although cooks were routinely trained, the characteristics of the food and beverages in the hospital did not comply with the IDDSI nomenclature.

#### Forms of recording

To ensure the accuracy and reproducibility of the results, recordings were made for all samples using a recording protocol developed by the researcher. For each sample, the IDDSI values and volume remaining in the syringe after the flow test were recorded, allowing the classification of food consistency. Additionally, the log recorded the temperature of the samples at two different times: after meal preparation and one hour later (Material suplementar 1).

#### Statistical analysis

The Shapiro–Wilk normality test was used to compare the temperature and remaining liquid in the syringe at T0 and T1 for all samples. For data with a normal distribution, the Paired Student's t-test was applied, and for data that did not show a normal distribution, the Wilcoxon test was used. The assumed significance level was set at p < 0.05.

The Shapiro-Wilk normality test and Kruskal-Wallis test with Dunn's post hoc test were used for the statistical analysis of the interference of different cooks. The significance level was set at p < 0.05.

## RESULTS

As shown in Table 1, the measured temperature changed significantly after a one-hour interval for all food samples (porridge, pureed light soup, liquid soup, and smoothie).

**Table 1:** Comparison of the temperature (t) at Time 0 (T0) and Time 1 (T1) of all samples[[Q6: Q6]]

**Samples**	**T0 Mn (sd)**	**T1 Mn (sd)**	**P value**
Porridge	46.50 (13.65)	28.30 (3.18)	0.001*
Pureed light soup	47.13 (6.54)	36.6 (4.94)	0.000*
Liquid soup	4.97 (3.55)	38.17 (2.15)	0.000*
Smoothie	25.17 (1.09)	26.24 (0.45)	0.027*

* Significant values at P <0.05

**Caption:** Initial time (T0); Time after 1 hour (T1); Mean (Md); Standard deviation (sd)

However, when analyzing the remaining liquid in the syringe, it was found that in addition to the change in temperature, the one-hour interval also led to a change in the remaining volume in the porridge sample (P = 0.043), indicating a change in the IDDSI value from 3 to 4. Therefore, the temperature variation resulting from the time between preparation and consumption by the patient led to a change in the consistency of the porridge (IDDSI from 3 to 4). This change could pose a risk to the safety and efficiency of swallowing in hospitalized patients. For pureed light soup, liquid soup, and smoothies, no statistically significant differences were found in the changes in IDDSI values (Table 2).

**Table 2:** Comparison of Liquid Remaining (LR) data from samples in Time 0 (T0) and Time 1 (T1)

**Samples**	**LR T0**		**LR T1**		**P value**
	**Mn (sd)**	**Med (min-max)**	**Mn (sd)**	**Med (min-max)**	
+Porridge	9.14 (1.25)	9.90 (6.8-10)	9.61 (0.58)	10 (8.8-10)	0.043*
+Pureed light soup	9.87 (0.25)	10 (9.2-10)	9.96 (0.07)	10 (9.8-10)	0.48
Liquid soup	8.14 (1.45)	7.90 (6.2-9.8)	7.62 (2.36)	8.4 (3.8-10)	0.549
Smoothie	9.86 (0.13)	9.90 (9.6-10)	9.87 (0.04)	9.90 (9.6-10)	0.842

+ Wilcoxon test; Student Test T Paired the others

* Significant values at P < 0.05

**Caption:** Liquids Remaining in the syringe (LR); Initial Time (T0); Time after 1 hour (T1); Mean (Mn); Standard deviation (sd); Median (Med); Minimum-Maximum (min-max)

Table 3 shows differences in the remaining liquid in the syringe and consequently in the IDDSI value when analyzing samples prepared by different cooks. The changes occurred in the porridge (p = 0.021) and the smoothie (p = 0.045), indicating that the different cooks altered the textures of the porridge and the smoothie. Dunn's post hoc test revealed that for the porridge sample, the texture change occurred between cook 1 and cook 2 (p = 0.048) and between cook 2 and cook 3 (p = 0.048), changing from level 4 to 3. Thus, different kitchen teams influenced the preparation of the porridge. In the smoothie sample, there was a change between cook 2 and cook 3 (p = 0.049), with the smoothie changing from level 3 to 4.

**Table 3:** Analysis of food prepared by different chefs

**Samples**	**Cook 1**	**Cook 2**	**Cook 3**	**P**	**P adjusted**		
Cook 1-2						Cook 2-3	Cook 3-1
Porridge	10 (10-10)	8.8 (8.8-8.9)	10 (10-10)	0.021*	0,048*	0.048*	1
Pureed liquid soup	9.9 (9.8-10)	10 (10-10)	10 (10-10)	0.105			
Liquid soup	4.2 (3.6-7.6)	8.4 (6.4-9.6)	10 (8.6-10)	0.057			
Smoothie	9.9 (9.8-9.9)	9.8 (9.6-9.9)	10 (10-10)	0.045*	1	0.049*	0.224

Kruskal-Wallis Test; Dunn’s Post Hoc Test.

* Significant values at p < 0.05

**Caption:** Median (Minimum-Maximum); P value (P); Chefs (Chef.); Groups (P adjusted).

**Figure 4:**
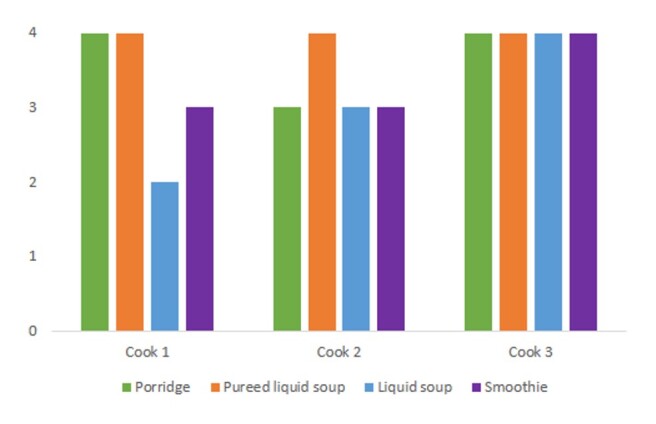
IDDSI levels, in triplicates, prepared by three cooks

Figure 4 shows triplicate measurements of the IDDSI values for thickened liquids: porridge, pureed light soup, liquid soup, smoothie, and preparation on three different days. The consistency of the meals varied depending on the IDDSI values of the different cooks.

## DISCUSSION

Our first hypothesis was confirmed, as temperature affected the flowability of the foods studied, with the IDDSI value for porridge changing from 3 at time zero (T0) to a value of 4 after one hour (T1). This phenomenon can be attributed to the 60 min that had passed since the end of preparation. Even a period of 30 min affected the temperature and consistency of the food, thus increasing the risk of complications during ingestion in patients with dysphagia^(17)^.

It is important to emphasize that in the hospital in the present study, food was stored and transported using non-thermal equipment. Proper storage can help control the temperature of food consumed by hospitalized patients. Therefore, it is crucial for the speech-language pathologist, who plays a role in hospital management or collaborates with administration, to seek resources and strategies to demonstrate the importance of maintaining food temperature, ensuring the safety and efficiency of swallowing in patients with dysphagia^(17)^. Even if the prepared food reaches an appropriate temperature at the end of the cooking process, temperature variations between the maintenance and final distribution phases may represent critical periods for the proliferation of microorganisms^(18)^. Although this aspect was not analyzed in our study, it should be taken into account since hospitalized patients generally have low immunity, and any contamination can compromise recovery^(19)^.

Although a temperature change occurred in all foods tested, analysis of the remaining liquid in the syringe showed that only the porridge sample changed the IDDSI value from 3 to 4, whereas no change in the value was observed in the other foods. Changes in food consistency affect swallowing management, particularly in hospitalized patients with swallowing difficulties who require speech therapy^(20)^.

Several studies have used standardization methods^(21)^ such as the IDDSI to classify foods and beverages in patients with dysphagia^(22,23)^. However, few studies have controlled for the variables that may influence food management. There is also insufficient research on the effects of different food consistencies on food processing during the oral preparation phase of swallowing^(24)^.

Our second hypothesis was partially confirmed, as the change in kitchen staff affected the consistency of the food (porridge) and drink (smoothie), resulting in a change from stage 4 to 3 and from stage 3 to 4, respectively. Therefore, the method of food preparation by different cooks may significantly affect the texture of prepared foods and consequently affect the stages proposed by the IDDSI^(17)^.

We conducted research using both experimental and benchmark methods. However, it is worth noting that the recommendations for grading consistency should be performed using the flow test before offering food to the patient^(9)^. In clinical practice, speech-language pathologists face challenges such as the need to manipulate food and beverages offered to patients. Therefore, the control of external variables, such as temperature and the continuous training of professionals responsible for food preparation, together with collaboration with the hospital's nutrition department, favors the preparation of food with better consistency.

It should be emphasized that kitchen teams in hospitals may also face some difficulties when preparing meals, such as achieving the right consistency of food for hospitalized patients, because the lack of appropriate equipment and ingredients may hinder the achievement of the right consistency^(17)^.

The speech therapist is responsible for the assessment, diagnosis, and intervention of dysphagia symptoms, as well as for educating the multidisciplinary team on the changes in swallowing physiology^(25)^, with the aim of ensuring appropriate food management so that consistencies remain safe for patients. This in turn reduces the risk of complications associated with aspiration pneumonia, leading to longer hospital stays and higher medical costs^(26)^.

Therefore, it is important that healthcare professionals are aware of the consequences of dysphagia^(27)^ and that kitchen teams are properly trained in food preparation to minimize errors in the hospital and ensure patient safety^(28)^. The implementation of these practices can lead to significant improvements in dysphagia management and patient quality of life^(27)^.

## LIMITATIONS OF THE STUDY

One limitation of the present study is that the flow test was not performed with thermally stored food, as this did not reflect the reality in the hospital under investigation. Additionally, industrially thickened foods were not evaluated.

## CONCLUSION

The temperature of all diets varied within one hour, leading to changes in the flow test and IDDSI values, specifically from level 3 to level 4 for the porridge. Different chefs prepared smoothies and porridges with varying characteristics, resulting in changes from level 3 to level 4 for both samples.

## Supplementary Material

Supplementary material accompanies this paper.

Material suplementar 1 Protocolo de registro da coleta de dados.

This material is available as part of the online article from https://doi.org/10.1590/2317-1782/20242023315en
