# Supplementary material for: The influence of temperature variation on the levels of the International Dysphagia Diet Standardization Initiative
Source: Codas. 2024 Sep 13;36(6):e20230315. doi: 10.1590/2317-1782/20242023315en (PMC11405055; doi:10.1590/2317-1782/20242023315en)
Supplement: Material suplementar 1 [file codas-36-6-e20230315-Suppl.pdf]

Material suplementar 1: Protocolo de registro da coleta de dados

Tempo: 0
Data:
Período:
Nome do cozinheiro:

**Cardápio**	**Amostra 1**				**Amostra 2**				**Amostra 3**			
	Temp	Volume seringa	Nível	Hora	Temp	Volume seringa	Nível	Hora	Temp	Volume seringa	Nível	Hora
Mingau												
Sopa leve batida												
Sopa líquida												
Vitamina												

Tempo: 1
Data:
Período:
Nome do cozinheiro:

**Cardápio**	**Amostra 1**				**Amostra 2**				**Amostra 3**			
	Temp	Volume seringa	Nível	Hora	Temp	Volume seringa	Nível	Hora	Temp	Volume seringa	Nível	Hora
Mingau												
Sopa leve batida												
Sopa líquida												
Vitamina												
