# Supplementary material for: The influence of temperature variation on the levels of the International Dysphagia Diet Standardization Initiative
Source: Codas. 2024 Sep 13;36(6):e20230315. doi: 10.1590/2317-1782/20242023315en (PMC11405055; doi:10.1590/2317-1782/20242023315en)
Supplement: Translation

# Translation: A interferência da variação da temperatura nos níveis da Iniciativa Internacional de Padronização de Dietas para Disfagia

**DOI:** 10.1590/2317-1782/20242023315pt

**Authors:** Tainá Cristina do Espírito Santo, Adriana Ponsoni, Cinthia Madeira de Souza, Lucia Figueiredo Mourao

**Affiliations:** 1 Faculdade de Ciências Médicas, Universidade Estadual de Campinas – UNICAMP - Campinas (SP), Brasil.; 2 Programa de Pós-graduação em gerontologia, Faculdade de Ciências Médicas, Universidade Estadual de Campinas – UNICAMP - Campinas (SP), Brasil.; 3 Departamento de Desenvolvimento Humano e Reabilitação, Faculdade de Ciências Médicas, Universidade Estadual de Campinas – UNICAMP -Campinas (SP), Brasil.

**Keywords:** Deglutição, Transtorno da Deglutição, Viscosidade, Temperatura, Hospitais

## Abstract

**Objetivo:**

1) Analisar a influência da temperatura no fluxo/textura dos diferentes alimentos, logo após o preparo e após uma hora, e 2) Comparar a influência de variar o cozinheiro no preparo dos alimentos, em relação ao fluxo dos alimentos.

**Método:**

Trata-se de um estudo quantitativo e experimental. Utilizou-se o teste de fluxo padronizado pelo IDDSI, para avaliar o volume restante da seringa e os níveis dos alimentos (mingau, vitamina, sopa líquida e sopa leve batida) preparados por diferentes cozinheiros, em triplicata, no tempo zero (T0) e após uma hora (T1).

**Resultados:**

Foi observado diferenças na temperatura em todos os alimentos no T0 e T1 (p < 0,05). O nível do IDDSI mudou apenas no mingau, de nível 3 para 4 (p = 0,043). Modificações foram observadas no preparo por diferentes cozinheiros para vitamina, no 2° e 3° dia (p=0,049), do nível 3 para 4 do IDDSI. No mingau, no 1° e 3°dia (p=0,048) e 2°e 3° dia (p=0,048), com mudança de nível 4 para 3 do IDDSI.

**Conclusão:**

A temperatura de todos os alimentos foi diferente, no intervalo de uma hora, com modificações no teste de fluxo e nos níveis do IDDSI, do nível 3 para 4, apenas para o mingau. Diferentes cozinheiros prepararam a vitamina e o mingau com características diferentes, e consequente, mudanças do nível 3 para 4 em ambos os alimentos.

## INTRODUÇÃO

A deglutição é um processo complexo e vital que envolve estruturas correlacionadas entre si e com mecanismos neuronais^(1)^. Alterações na neurofisiologia da deglutição ocasionam a disfagia, que é caracterizada como dificuldade na preparação do bolo alimentar ou em seu deslocamento até o estômago^(2)^. A disfagia, quando presente no ambiente hospitalar, contribui para o aumento dos custos com o cuidado da saúde e prolonga o tempo de internação^(3)^, resultando na sobrecarga do sistema de saúde^(3)^. A prevalência da disfagia é maior em pacientes hospitalizados^(4)^ do sexo masculino, portadores de doenças cardíacas e idosos^(4)^, afetando cerca de 50% deste último grupo^(5)^. As causas subjacentes são doenças neurológicas e o envelhecimento de estruturas relacionadas ao processo de deglutição^(6)^, resultando em desnutrição, desidratação, aumento do tempo de internação, complicações respiratórias com necessidade de intubação, tratamento medicamentoso para pneumonia aspirativa, até morte^(5)^.

No ambiente hospitalar o manejo da disfagia, prioritariamente, visa proporcionar a deglutição segura e eficiente^(7,8)^, uma das opções terapêuticas comumente adotadas é a modificação das consistências dos alimentos^(8)^ e líquidos, para facilitar a deglutição e reduzir os riscos de broncoaspiração^(9,10)^. A utilização de modificações das texturas alimentares, como forma de intervenção clínica^(8)^, levou à necessidade de estabelecer uma padronização internacional para classificar todas as consistências alimentares^(11)^. Em 2013, a Iniciativa Internacional de Padronização de Dietas para Disfagia (IDDSI)^(12)^ desenvolveu uma terminologia para classificação das texturas de alimentos e espessura das bebidas por meio de um diagrama (diagrama IDDSI), que proporciona aos profissionais a indicação adequada do alimento ou bebida para o indivíduo disfágico. Além disso, favorece uma comunicação eficiente entre o fonoaudiólogo e a equipe multidisciplinar^(8)^ . Os testes do IDDSI são constituídos por testes simples e de fácil acesso, que devem ser realizados nas condições nas quais os alimentos e bebidas irão ser servidos (principalmente a temperatura)^(9)^. Um estudo descreveu que a viscosidade e temperatura, são fatores que interferem na consistências dos alimentos^(13)^. Ao adicionar espessantes à base de amido de milho ao café a 70ºC, têm-se como resultado uma amostra mais espessa, quando comparada ao preparo com temperatura ambiente (25ºC), isso ocorre porque os grânulos do amido de milho aumentaram a viscosidade do líquido durante o processo de geleificação^(13)^. Desta forma, essas variáveis têm o potencial de ocasionar alterações na consistência dos fluidos, o que, por sua vez, pode comprometer a segurança da deglutição em pacientes com disfagia^(14)^.

Assim, as hipóteses do estudo foram que a variação da temperatura dos alimentos, desde o momento de finalização do preparo até a sua oferta ao paciente, pode sofrer modificações. Nossa segunda hipótese é que diferentes cozinheiros podem apresentar preparo das refeições de modo diferente, com impacto no fluxo de líquidos-engrossados. Desta forma, os objetivos foram: 1) Analisar a influência da temperatura no fluxo/textura dos diferentes alimentos, logo após o preparo e após uma hora, e 2) Comparar a influência de variar o cozinheiro no preparo dos alimentos, em relação ao fluxo dos alimentos.

## MÉTODO

### Delineamento do estudo

O presente estudo foi desenvolvido com metodologia de natureza quantitativa e de abordagem experimental. Cabe salientar que a pesquisa experimental se caracteriza por manipular diretamente as variáveis relacionadas com o objeto de estudo e tem como finalidade testar hipóteses^(15)^. A manipulação na quantidade e qualidade das variáveis proporciona relação entre a causa e o efeito, sendo necessário controlar a variável e avaliar os resultados dessas relações^(16)^. Portanto, nossas variáveis dependentes foram: a temperatura e a participação de diferentes cozinheiros no preparo das refeições.

### Local e período da coleta

A coleta dos dados ocorreu no Hospital de Clínicas da Universidade Estadual de Campinas (Unicamp), no período de agosto de 2022. Por se tratar de uma pesquisa experimental de bancada sem a intervenção com seres humanos, não foi necessário submeter ao Comitê de Ética.

### Materiais do estudo

Os materiais utilizados na coleta de dados foram: termômetro digital para aferir a temperatura dos alimentos, uma câmera de celular montada em um tripé para registrar todos os procedimentos através de filmagem, seringas de bico da marca BD® de 10 mL (padrão para o teste de fluxo) e utensílios de plástico, como colher e garfo.

### Instrumentos da pesquisa

#### Teste de fluxo

O instrumento utilizado para a coleta de dados foi o teste de fluxo (Figura 1), no qual os líquidos espessados foram inseridos em uma seringa BD (10 mL) até a marcação de 10 mL. Em seguida, o bico da seringa foi coberto com o dedo para evitar o escoamento do líquido. Juntamente com a liberação do dedo, foi cronometrado o tempo de 10 segundos e, ao finalizar a contagem, o bico foi ocluído (Figura 1). Desta forma, verificou-se a quantidade remanescente do fluido na seringa^(12)^.

**Figura 1:** Teste de fluxo do IDDSI

Os líquidos remanescentes foram classificados de acordo com os níveis de 0 a 4 do IDDSI: Nível 0 - Líquido ralo, menos de 1 mL de líquido remanescente na seringa ao final dos 10 segundos de fluxo; Nível 1 - Muito levemente espessado, entre 1 a 4 mL remanescentes na seringa; Nível 2 - Levemente espessados, entre 4 a 8 mL remanescentes na seringa; Nível 3 - Moderadamente espessado, considera-se quando há mais de 8 mL remanescentes na seringa^(12)^.

No nível 4 - Extremamente espessado. O teste de fluxo não é aplicável (10 mL remanescentes), sendo realizado o teste de inclinação de colher ou gotejamento e pressão do garfo^(12)^ .

No presente estudo realizou-se os testes de inclinação de colher, gotejamento ou pressão do garfo por meio da metodologia validada pelo IDDSI^(12)^. O primeiro consistiu na inclinação da colher com o alimento, a qual deve cair sobre a superfície e sobrar pouco ou nenhum resíduo no utensílio^(12)^. No segundo teste, foi necessário colocar o alimento sobre o garfo e verificar o gotejamento^(12)^. No último, pressionou-se o garfo sobre o alimento e para classificação foi necessário a observação de discretas marcas do talher^(12)^.

#### Coleta de dados

A coleta de dados do estudo ocorreu em duas etapas, sendo a primeira a verificação da temperatura e a segunda etapa a variação dos cozinheiros no preparo dos alimentos. Por se tratar de um estudo experimental de bancada e para garantir a fidedignidade, precisão, confiabilidade e reprodutibilidade dos resultados, com o objetivo de minimizar erros experimentais, nas Etapa 1 e 2 (Figuras 2, 3), a coleta foi realizada em triplicatas, isto é, cada variável que pudesse influenciar na mudança da textura dos alimentos, foram testadas três vezes.

**Figura 2:** Demonstração das coletas de todos os alimentos, em triplicata, no Tempo 0 (T0) e Tempo 1 (T1)

**Figura 3:** Demonstração da coleta dos alimentos em triplicadas, com a variação dos cozinheiros

Devido ao estudo ser um delineamento experimental foi selecionada a amostra de cada um dos alimentos estudados com a menor variação da variável dependente (temperatura e cozinheiro).

#### Etapa 1 - Temperatura

Nessa primeira etapa do teste de fluxo do IDDSI, foram coletadas as temperaturas dos alimentos. Inicialmente, a pesquisadora aferiu a temperatura com o termômetro digital, após o preparo de cada refeição (mingau, sopa leve batida, sopa líquida e vitamina) (Quadro 1), designado de tempo zero (T0), e uma hora depois, referido como tempo um (T1). O intervalo de uma hora foi escolhido com o intuito de reproduzir o tempo estimado para que a refeição chegasse ao leito dos pacientes hospitalizados.

**Quadro 1:** Caracterização dos alimentos

**Alimentos**	**Características**	**Ingredientes**	**Calorias (kcal)**	**Proteínas (g)**	**Carboidratos (g)**	**Lipídeos (g)**
Mingau	Ingredientes triturados	Leite, açúcar e amido de milho	248,72	8,31	35,09	8,3
Sopa leve batida	Ingredientes triturados	Água, cenoura, proteína animal e temperos	302,51	16,4	33,78	11,3
Sopa líquida	Ingredientes triturados e mais espessa que água	Água, cenoura, proteína animal e temperos	302,51	16,4	33,78	11,3
Vitamina	Ingredientes triturados	Leite, banana e groselha	333,14	8,2	72,2	4,63

**Legenda:** Quilocaloria (kcal); gramas (g)

A temperatura dos alimentos (mingau, sopa leve batida, sopa líquida e vitamina) foram aferidos a temperatura no T0 e T1 (Figura 2), em três amostras de cada um dos alimentos, ou seja em triplicadas sendo considerado a menor variação da temperatura para a análise.

#### Etapa 2 – Análise de diferentes cozinheiros na preparação dos alimentos

Durante um período de três dias alternados, os alimentos (mingau, sopa leve batida, sopa líquida e vitamina) também foram coletadas em triplicatas. Os dias alternados foram selecionados com o objetivo de incluir diferentes cozinheiros no estudo, devido à rotatividade dos funcionários na preparação das refeições. Assim, na segunda fase do estudo, os cozinheiros que preparam as refeições foram considerados como variáveis dependentes (Figura 3). 

Cabe ressaltar que, durante o preparo das refeições, os diferentes cozinheiros utilizaram receitas padronizadas, com quantidades idênticas dos ingredientes, definidas pela equipe de nutrição do hospital. Todos os ingredientes industrializados eram da mesma marca. Os cozinheiros são treinados rotineiramente, mas as características dos alimentos e bebidas do referido hospital não utilizam a nomenclatura IDDSI.

#### Formas de registro

A fim de garantir a precisão e a reprodutibilidade dos resultados, foram realizadas filmagens de todos os testes das amostras, juntamente com um protocolo de registro que foi elaborado pela pesquisadora. Foram anotados os níveis do IDDSI e o volume restante da seringa após o teste de fluxo para cada amostra, permitindo a classificação da consistência dos alimentos. Além disso, o protocolo também registrava a temperatura das amostras em dois momentos distintos, após o preparo das refeições e uma hora depois (Material suplementar 1).

#### Análise estatística

Primeiramente, para comparar a temperatura e também os líquidos remanescentes na seringa no T0 e T1 de todas as amostras, utilizou-se o teste de normalidade Shapiro-Wilk. Para dados com distribuição normal foi aplicado o teste T Student Pareado e para os dados que não apresentaram distribuição normal, foi utilizado o Teste Wilcoxon. O nível de significância adotado foi p < 0,05.

Para a análise estatística da comparação da interferência dos diferentes cozinheiros, utilizou-se o teste de normalidade Shapiro-Wilk e o teste Kruskal-Wallis, com Post-Hoc de Dunn. O nível de significância estabelecido foi p < 0,05.

## RESULTADOS

Conforme observado na Tabela 1, a temperatura aferida com intervalo de uma hora se modificou em todas as amostras dos alimentos (mingau, sopa leve batida, sopa líquida e vitamina), de modo estatisticamente significantes.

**Tabela 1:** Comparação da temperatura (t) no Tempo 0 (T0) e Tempo 1 (T1) de todas as amostras

**Amostras**	**T0 Md (dp)**	**T1 Md (dp)**	**P valor**
Mingau	46,50 (13,65)	28,30 (3,18)	0,001^*^
Sopa leve batida	47,13 (6,54)	36,6 (4,94)	0,000*
Sopa líquida	46,97 (3,55)	38,17 (2,15)	0,000*
Vitamina	25,17 (1,09)	26,24 (0,45)	0,027*

* Valores significativos a P <0,05

**Legenda:** Tempo inicial (T0); Tempo após 1 hora (T1); Média (Md); Desvio Padrão (dp)

Entretanto, quando realizada a análise do líquido remanescente da seringa, foi possível evidenciar que além da mudança na temperatura, o intervalo de uma hora resultou em modificação no volume restante da seringa na amostra de mingau (P = 0,043), ou seja, ocorreu a mudança do nível do IDDSI de 3 para 4. Desta forma, a variação da temperatura resultado do tempo que leva do final da preparação até o consumo pelo paciente, ocasionou a modificação da consistência do alimento mingau (IDDSI de 3 para 4). A mudança de nível pode levar a riscos na segurança e eficiência da deglutição do paciente hospitalizado. Não foram observadas diferenças estatisticamente significantes na modificação dos níveis do IDDSI para as refeições sopa leve batida, sopa líquida e vitamina (Tabela 2).

**Tabela 2:** Comparação dos dados dos Líquidos Remanescentes (LRS) das amostras no Tempo 0 (T0) e no Tempo 1 (T1)

**Amostras**	**LRS T0**		**LRS T1**		**P valor**
	**Md (dp)**	**Med (min-máx)**	**Md (dp)**	**Med (min-máx)**	
+Mingau	9,14 (1,25)	9,90 (6,8-10)	9,61 (0,58)	10 (8,8-10)	0,043^*^
^+^ Sopa leve batida	9,87 (0,25)	10 (9,2-10)	9,96 (0,07)	10 (9,8-10)	0,48
Sopa líquida	8,14 (1,45)	7,90 (6,2-9,8)	7,62 (2,36)	8,4 (3,8-10)	0,549
Vitamina	9,86 (0,13)	9,90 (9,6-10)	9,87 (0,04)	9,90 (9,6-10)	0,842

+ Teste Wilcoxon; Teste T Student Pareado os demais

* Valores significativos a P < 0,05

**Legenda:** Líquidos Remanescentes na seringa (LRS); Tempo inicial (T0); Tempo após 1 hora (T1); Média (Md); Desvio Padrão (d); Mediana (Med); Mínimo-Máximo (mín-máx)

A Tabela 3 mostrou que ocorreram diferenças no líquido remanescente da seringa e, consequentemente, do nível do IDDSI, ao analisar a amostra preparada por diferentes cozinheiros. As mudanças ocorreram para o mingau (p=0,021) e a vitamina (p=0,045), ou seja, a variação do cozinheiro resultou em modificação das texturas do mingau e da vitamina. Com a realização do teste Post Hoc de Dunn, observou-se que na amostra de mingau a modificação da textura do alimento, ocorreu entre o cozinheiro 1 e o cozinheiro 2 (p=0,048) e entre o cozinheiro 2 e o 3 (p=0,048), com a mudança do nível 4 para o 3. Desta forma, as diferentes equipes da cozinha interferiram na preparação do mingau. Na amostra de vitamina ocorreu variação entre o cozinheiro 2 e o 3 (p=0,049), a vitamina passou do nível 3 para o 4.

**Tabela 3:** Análise dos alimentos preparados pelos diferentes cozinheiros

**Amostras**	**Cozinheiro 1**	**Cozinheiro 2**	**Cozinheiro 3**	**P**	**P ajustado**		
Coz. 1-2						Coz. 2-3	Coz. 3-1
Mingau	10 (10-10)	8,8 (8,8-8,9)	10 (10-10)	0,021*	0,048*	0,048^*^	1
Sopa leve batida	9,9 (9,8-10)	10 (10-10)	10 (10-10)	0,105			
Sopa líquida	4,2 (3,6-7,6)	8,4 (6,4-9,6)	10 (8,6-10)	0,057			
Vitamina	9,9 (9,8-9,9)	9,8 (9,6-9,9)	10 (10-10)	0,045*	1	0,049*	0,224

Teste Kruskal-Wallis; Teste Post Hoc de Dunn. *Valores significativos a p < 0,05

**Legenda:** Mediana (Mínimo-Máx); P valor (P); Cozinheiros (Coz.); Grupos (P ajustado).

Observou-se na Figura 4, as medidas em triplicata dos níveis do IDDSI dos líquido-espessados: mingau, sopa leve batida, sopa líquida e vitamina, além da realização em três dias diferentes de preparo. Nota-se que as consistências das refeições sofreram modificações, de acordo com os níveis do IDDSI, entre os diferentes cozinheiros.

**Figura 4:** Níveis IDDSI, em triplicatas, preparadas por três cozinheiros

## DISCUSSÃO

A nossa primeira hipótese foi confirmada, uma vez que a temperatura influenciou no fluxo dos alimentos estudados, com nível IDDSI 3 no tempo zero para o nível 4 após uma hora, no caso do mingau. Tal fenômeno pode ser atribuído ao período de 60 minutos decorrido do término do preparo. O tempo de 30 minutos já interferiu na temperatura e modificações das consistências dos alimentos, aumentando assim o risco de complicações durante a alimentação de pacientes com disfagia^(17).^

É importante ressaltar que, no hospital do presente estudo, os alimentos são armazenados e transportados em equipamentos não térmicos. Acredita-se que o acondicionamento adequado, pode favorecer o controle da temperatura dos alimentos consumidos pelos pacientes hospitalizados. Desta forma, um fator relevante é que caberá ao fonoaudiólogo, que desempenha seu papel na gestão hospitalar ou em colaboração com a administração, procurar recursos e estratégias com o objetivo de demonstrar a importância da manutenção da temperatura dos alimentos, a fim de garantir a segurança e eficiência da deglutição do paciente com disfagia^(17)^. Ainda que os alimentos preparados atinjam temperaturas adequadas no final do cozimento, a mudança de temperatura desde o período de manutenção até a distribuição final pode representar períodos críticos para a proliferação de microrganismos^(18)^. Este é um aspecto que não foi analisado em nosso estudo, mas deve ser considerado, dado que os pacientes hospitalizados geralmente têm baixa imunidade e qualquer contaminação pode interferir na recuperação^(19)^.

Embora, a mudança da temperatura tenha ocorrido para todos os alimentos testados, ao analisar o líquido remanescente na seringa, evidenciou-se que somente na amostra do mingau ocorreu a mudança de nível do IDDSI de 3 para 4, não sendo observado a variação do nível para outros alimentos. A modificação da consistência dos alimentos interferem no manejo da deglutição, especialmente para pacientes hospitalizados com dificuldades de deglutição que necessitam de intervenção fonoaudiológica^(20).^

Diversos estudos têm empregado formas de padronizações^(21)^, como o IDDSI, para a classificação dos alimentos e bebidas para o paciente disfágico^(22,23)^. No entanto, a literatura apresenta poucos estudos sobre o controle das variáveis que podem influenciar no manejo dos alimentos. Além do que, o impacto de diferentes consistências no processamento oral dos alimentos durante a fase preparatória oral da deglutição também é pouco explorado^(24)^.

Nossa segunda hipótese, foi parcialmente confirmada, pois a mudança da equipe da cozinha interferiu na consistência dos alimentos (mingau) e bebida (vitamina), ocasionando mudanças no nível 4 para 3 e 3 para 4, respectivamente. Desta forma, o modo de preparo dos alimentos por diferentes cozinheiros pode influenciar significativamente na textura dos alimentos preparados, interferindo como consequência nos níveis propostos pelo IDDSI^(17)^.

Nosso estudo realizou uma pesquisa com metodologia experimental e de bancada. Entretanto, cabe ressaltar que as recomendações para a classificação dos níveis de consistência dos alimentos por meio do teste de fluxo devem ser realizadas antes da oferta para o paciente^(9)^. Na prática hospitalar, o fonoaudiólogo enfrentará desafios, como a necessidade de manipular novamente os alimentos e bebidas que serão oferecidos aos pacientes. Portanto, o controle das variáveis externas, como temperatura, e a educação continuada dos profissionais responsáveis pelo preparo dos alimentos, juntamente com a colaboração do setor de nutrição do hospital, favorecem uma preparação com consistências mais adequadas dos alimentos.

Ressalta-se que as equipes de cozinha nos ambientes hospitalares também podem enfrentar algumas dificuldades para a preparação das refeições, como por exemplo, alcançar a consistência correta dos alimentos aos pacientes hospitalizados, pois a falta de equipamentos – e também de ingredientes – adequados podem dificultar na obtenção das texturas apropriadas^(17)^.

O fonoaudiólogo é o profissional responsável pela avaliação, diagnóstico e intervenção dos sintomas de disfagia, bem como pelo treinamento da equipe multidisciplinar sobre as alterações da fisiologia da deglutição^(25)^, visando garantir o manejo adequado dos alimentos para assegurar que as consistências permaneçam seguras para os pacientes. Isso, por sua vez, diminuirá o risco de complicações relacionadas à broncoaspiração, como a pneumonia aspirativa, que pode levar a internações hospitalares prolongadas e aumentar os custos médicos^(26)^.

Portanto, é essencial que os profissionais de saúde estejam cientes das consequências da disfagia^(27)^ e que as equipes de cozinheiros recebam treinamento adequado sobre a preparação dos alimentos, pois isso pode minimizar erros nos ambientes hospitalares e garantir a segurança do paciente^(28)^. A implementação dessas práticas pode levar a uma melhoria significativa na gestão da disfagia e na qualidade de vida dos pacientes^(27)^.

## LIMITAÇÕES DO ESTUDO

Uma limitação do presente estudo foi a não realização do teste de fluxo com os alimentos armazenados em transporte térmico, visto que essa não era a realidade do hospital em estudo. Também não foram avaliados alimentos com espessantes industrializados.

## CONCLUSÃO

A temperatura de todas as dietas foi diferente, no intervalo de uma hora, com modificações no teste de fluxo e nos níveis do IDDSI, do nível 3 para 4, apenas para o mingau. Diferentes cozinheiros prepararam a vitamina e o mingau com características diferentes, e consequente, mudanças do nível 3 para 4 em ambas as amostras.

## Material Suplementar

Este artigo acompanha material suplementar.

Material suplementar 1 Protocolo de registro da coleta de dados.

Este material está disponível como parte da versão online do artigo na página https://doi.org/10.1590/2317-1782/20242023315pt
